# Breastfeeding and vitamin D supplementation reduce the risk of Kawasaki disease in a German population-based case-control study

**DOI:** 10.1186/s12887-019-1438-2

**Published:** 2019-02-26

**Authors:** K. Meyer, A. Volkmann, M. Hufnagel, E. Schachinger, S. Klau, J. Horstmann, R. Berner, M. Fischer, A. Lehner, N. Haas, S. Ulrich, A. Jakob

**Affiliations:** 1 Department of Congenital Heart Disease and Pediatric Cardiology, University Heart Center Freiburg, Mathildenstraße 1, D-79106 Freiburg, Germany; 2 Institute for Medical Information Processing, Biometry and Epidemiology, Ludwig-Maximilians-University of Munich, Munich, Germany; 3 Division of Pediatric Infectious Disease and Rheumatology, Center for Pediatrics and Adolescent Medicine, University Medical Center Freiburg, Freiburg, Germany; 4 Department for Pediatrics, University Hospital Carl Gustav Carus of the Technische Universität Dresden, Dresden, Germany; 5 Department of Pediatric Cardiology, Ludwig-Maximilians-University of Munich, Munich, Germany

**Keywords:** Kawasaki disease, Coronary artery aneurysm, Risk factors, Vitamin D supplementation, Breast feeding

## Abstract

**Background:**

In Kawasaki disease (KD), a vasculitis of unknown etiology, the most serious complication is the development of coronary artery aneurysm (CAA). To date, the exact pathomechanism of KD is unknown. Both environmental and genetic factors seem to be associated with the development of the disease.

**Methods:**

Data on KD patients recruited from the population-based German Pediatric Surveillance Study during 2012–2014 were used to evaluate the impact of various factors from the perinatal and infancy period on the development of KD. The study design was a matched case-control study with respect to age, sex and place of residence (*n* = 308 KD cases, *n* = 326 controls). All KD patients were individually re-evaluated; all fulfilled the international diagnostic KD criteria. A standardized questionnaire was used to review breastfeeding practices, vitamin D supplementation and birth characteristics. Logistic regression analyses were performed to obtain odds ratios (OR) for various risk factors among the case-control pairs. Simple measures of association were used to assess the impact of these factors on the clinical course.

**Results:**

There was no difference in lengths of gestation, birth weight or parturition between KD patients and controls, but independently from each other vitamin D supplementation and breastfeeding were negatively associated with KD, even when adjusted for age, place of residence and sex. The duration of vitamin D was significantly shorter among children with KD than among children without KD (*p* = 0.039, OR = 0.964, 95% CI: 0.931–0.998), as was the duration of breastfeeding (*p* = 0.013, OR = 0.471, 95% CI: 0.260–0.853). Comparing KD patients with and without breastfeeding and/or vitamin D supplementation, there were no differences regarding developing CAA, being refractory to intravenous immunoglobulin treatment, age at onset of the disease and levels of inflammatory laboratory values.

**Conclusion:**

Our findings indicate breastfeeding and vitamin D supplementation to have protective effects in association with KD in our study population; however, these seem not to influence the natural course of the disease. Although the overall effects were relatively small, they nevertheless underline the overall benefit of both interventions.

**Trial registration:**

Clinical Trial Registration: German clinical trial registration, http://apps.who.int/trialsearch/Trial2.aspx?TrialID=DRKS00010071. Date of registration was 26. February 2016. The trial was registered retrospectively.

**Electronic supplementary material:**

The online version of this article (10.1186/s12887-019-1438-2) contains supplementary material, which is available to authorized users.

## Background

Kawasaki disease (KD) is a vasculitis of unknown etiology, which can be complicated by the development of coronary artery aneurysm (CAA). It is a rare disease, primarily affecting children under five years of age. The incidence among Asian children is much higher than among Caucasians. In our 2011–2012 cohort, we estimated an incidence of 7.2/100,000 in Germany (children < 5 years) [1, 2]. By contrast, for the year 2012 Japan reported the highest incidence worldwide, with 265/100,000 children (aged 0–4 years) diagnosed [3]. Cause and pathogenesis of KD are unclear. A combination of different risk factors, for disease development, along with a genetic predisposition, so far has been assumed [2, 4, 5]. To further investigate this assumption, we analysed the impact of different risk factors for KD during the perinatal period and infancy. Week of gestation, birth weight and type of parturition are known to impact health in later life [6]. Caesarean delivery, for example, is known to be a risk factor for multiple chronic immune diseases, including asthma and juvenile idiopathic arthritis [7]. Similarly, vitamin D supplementation and breastfeeding are well-known for their health benefits: In Germany, vitamin D supplementation generally is recommended for the first year of life. Besides playing an important role in the bone and calcium balance [8], vitamin D also regulates other cell functions and supports the immune system overall [9]. Furthermore, epidemiological studies on vitamin D deficiency show potential associations with cardiovascular diseases [10] and vasculitis [11].

For its health and other benefits, breastfeeding is preferable to formula feeding. Among other advantages, breastfeeding seems to protect against a variety of infectious diseases [12] and non-breastfed children have a higher risk of developing allergies and bronchial asthma later in life [13]. A Japanese study already has indicated a potential protective effect in KD [14].

During the perinatal and infancy periods, these and other factors potentially play a role in the pathogenesis of KD. Therefore, in a population-based, retrospective case-control study we investigated the association and impact of birth characteristics, breastfeeding practices and vitamin D supplementation on KD.

## Methods

Our study was designed as an epidemiological case-control study. KD cases were recruited from the population-based German Pediatric Surveillance Study (ESPED) from January 2012 to December 2014 [15]. For the ESPED report, pseudonymous identifiers were used. A standardized questionnaire was sent to reporting physicians requesting clinical and laboratory details from each KD case. Additionally, we requested that reporting physicians ask parents (via written informed consent) to agree to disclosure of their child’s identity so that our group could conduct follow-up questioning. In cases where such consent was provided, it became possible for us to validate the original ESPED data via discharge letters, recorded laboratory values and echocardiographic findings. For details see Additional file 1.

Due to data protection regulations, we only were able to include those KD cases where parents had provided written informed consent. Data regarding breastfeeding practices, vitamin D supplementation and birth characteristics were collected via a standardized questionnaire, and responses were provided online and in telephone interviews (see Additional file 2).

In order to obtain control cases from the KD patient’s own immediate environment, control cases (*n* = 326) were recruited from among the personal contacts of the KD case. First, the parents of KD cases were asked whether they might be able to recruit one of their child’s friends to take part in the survey. If no immediate friend of the KD cases was available or interested, then the treating paediatrician was asked to find a child who met the following criteria: (1) same sex, (2) same place of residence (≤ 50 km distance), (3) same age (≤ 6 months age difference) and (4) no medical history of KD. Once KD and control cases had been established, data were collected in an anonymous way through an online questionnaire. Case-control-pairing was conducted by using the ESPED pseudonymous identifier. In some cases, more than one control case was able to be recruited. For these KD cases, the control case was selected manually accordingly to best fit, which was based upon: (1) age, (2) gender and (3) place of residence. The best fitting case-control-pair was used for the statistical analysis.

All ESPED-reported KD cases (*n* = 631) were re-evaluated and checked for fulfilling the American Heart Association (AHA) guidelines for classification as either complete or incomplete KD cases [16]: Complete cases were defined as those with persistent fever for ≥5 days or fever that resolved within five days in response to intravenous immunoglobulin (IVIG) treatment. In addition, display of at least four principal clinical features was required: (1) changes in extremities; (2) polymorphous exanthema; (3) bilateral conjunctival injection without exudate; (4) enanthema of lips and oral cavity; and (5) bilateral cervical lymphadenopathy. By contrast, incomplete cases included either (A), (B) or (C) as outlined below:

(A) Those with fever (independent of age), plus fewer than four clinical features, plus detection of CAA. Diagnosis of CAA was based upon the clinical judgment of the reporting physician. In Germany, two criteria for CAA are applied: The first criterion used is from the Japanese Ministry of Health, which defines aneurysms either as a lumen > 3 mm in children under 5 years old, or as a diameter 1.5 times the size of the surrounding segment, or else as a clearly irregular lumen. The second criterion applied is a Z-score of above 2.5 for one of the coronary arteries.
(B) Those with fever under 6 months of age who have fewer than four clinical features.
(C) Those with fever over 6 months of age, plus three clinical features, plus laboratory evidence of systemic inflammation (CRP ≥30 mg/dl or ESR ≥40 mm/h) in combination with at least three of the following other abnormal supplemental laboratory findings: (1) increased alanine transaminase, (2) albumin ≤3.0 g/dL, (3) leukocyturia, (4) anaemia for age, (5) leukocytosis (≥15,000/mm^3^) and (6) thrombocytosis (≥450,000/mm^3^) [16].

In accordance with AHA guidelines, in cases where fever persists for over 36 h, German recommendations stipulate the administration of a second IVIG therapy course. Our study defined such cases as *refractory to IVIG*.

**Fig. 1:**
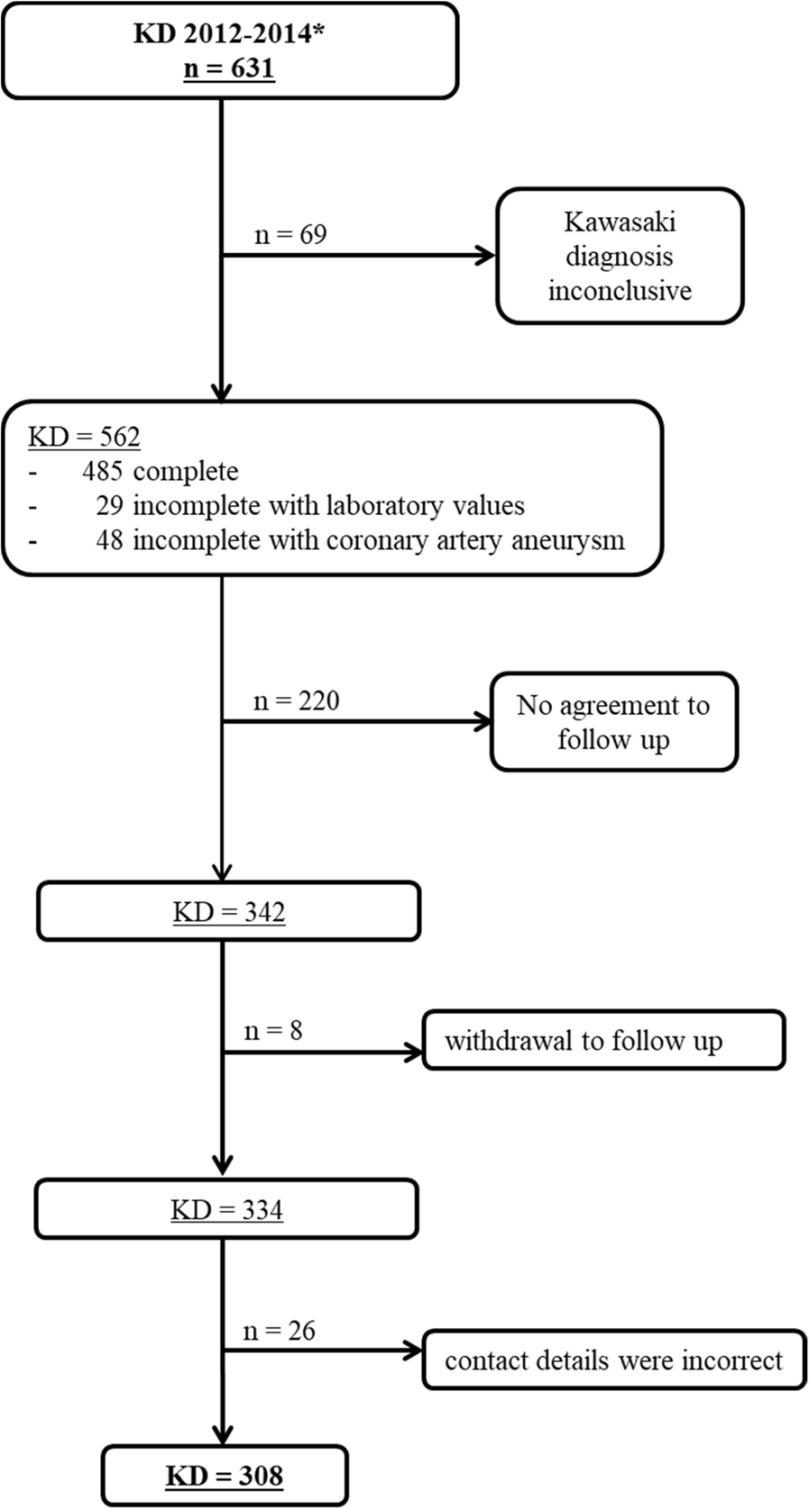
Study population. *2012 ≤ 4 years of age: *n* = 189, 2013/2014 ≤ 17 years of age: *n* = 442

Out of a total of *n* = 631 reported KD cases, *n* = 69 cases did not fulfil the AHA case definitions. Follow-up with complete data in response to our questionnaire was available in *n* = 308 cases. For details see Fig 1.

### Statistics

First, we analysed the quality of our matching (depending on the factors: age, place of residence and sex). Cross-tables, measures of association (phi coefficient, contingency coefficient, Cramer’s V and Pearson correlation coefficient) and OR were used. To identify possible risk factors for KD, we performed univariate analyses. For the main analysis, we employed a logistic regression conditional on the case-control pairs. As a sensitivity analysis, we also applied an unconditional logistic regression adjusting for the matching factors. The latter approach uses all available observations without restriction on successfully matched cases and controls; this results in a higher statistical power but can introduce some bias [17]. Therefore, the results of the conditional logistic regression were taken to represent our main results, while the results of the unconditional logistical regression were used as a sensitivity analysis to verify the main results.

All potential risk factors were analysed for associations with clinical findings (developing CAA, level of inflammatory laboratory values, age at onset of disease and being refractory to IVIG treatment). For this, we used the Chi-squared test, Spearman’s rank correlation coefficient and Cramer’s V. For breastfeeding and vitamin D supplementation, the age at onset of disease was examined using the Mann-Whitney test. Statistical analyses were performed using the statistical software R (3.3.2) and SPSS (24.0.0.0, IBM).

### Matching

**Fig. 2:**
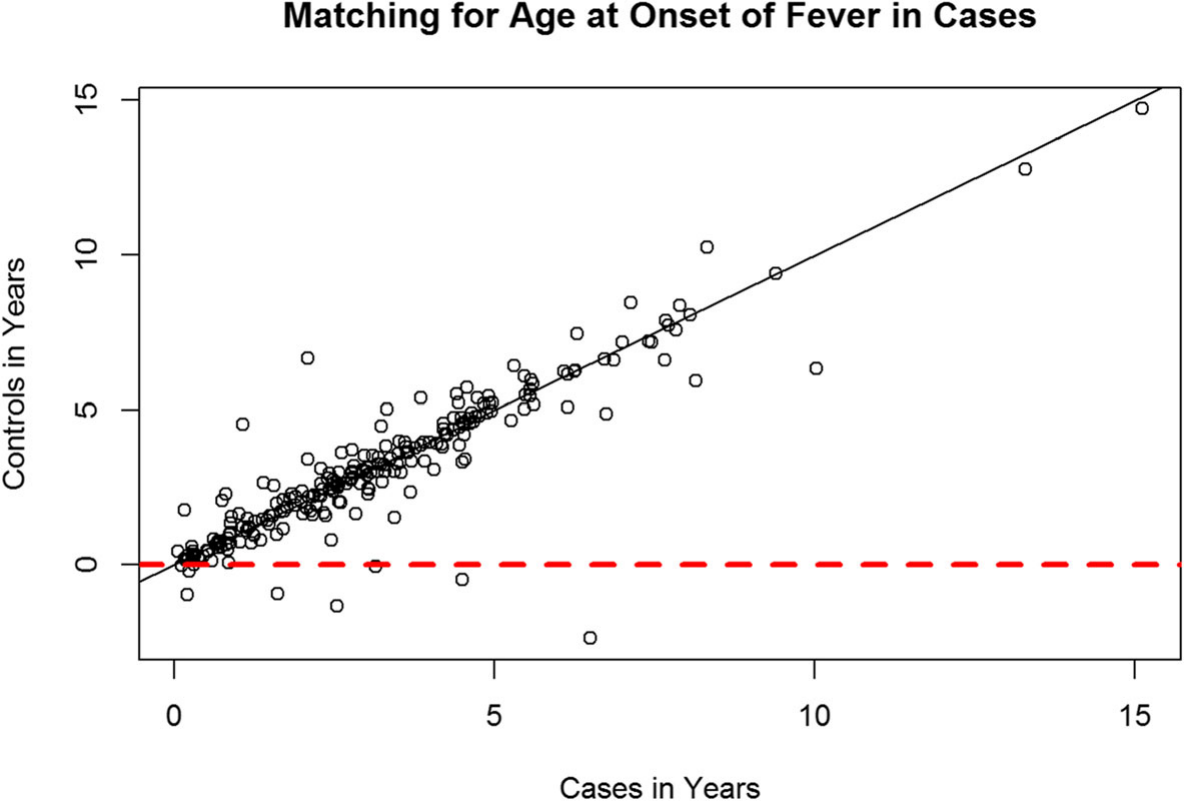
Scatterplot - Matching KD and control cases by age at the onset of fever in KD cases. One dot meets a matched pair. Dots that are below the red line were excluded. It is noticeable that most dots are on or next to the diagonal. Consequently, a successful matching based on age can be assumed

**Fig. 3:**
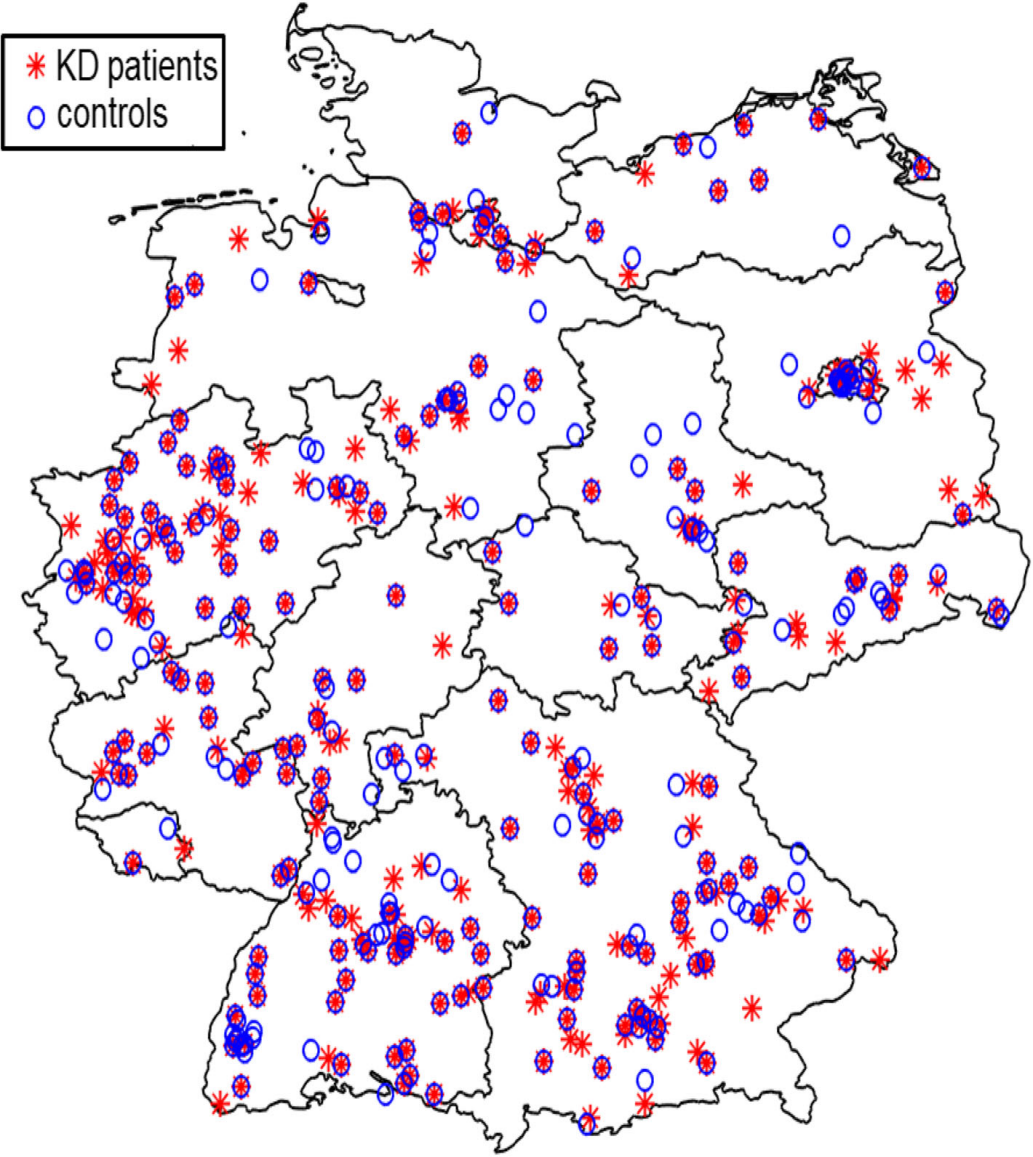
Matching: Geographical distribution of KD patients and their controls within Germany. We have created a map of Germany with the software MATLAB. Shapefiles, a file format containing geodata from the Database of Global Administrative Areas were used to extract the administrative boundaries in Germany. The individual postcodes were assigned to the respective federal state

A case-control matching was made based on sex, age and place of residence (Figs. 2, 3 and Table 1). The measures of association, i.e. contingency coefficient and Cramer’s V were > 0.9 for age and place of residence. This attests to the high quality of the matching and furthermore shows that the results would have been distorted, had the matching been ignored. With regard to sex, the measures of association were between 0.577 and 0.706, indicating a satisfactory case-control matching. Here, the OR for the matching factor sex was 36,346, meaning that the likelihood that a KD and control case would have the same sex was 36 times higher than that of their having different sexes.

**Table 1:** Measures of association for the matching factors sex, age and place of residence

	Matching factors		
	Sex	Age	Place of residence
Measures of association			
Phi coefficient	0.706		
Contingency coefficient	0.577	0.945	0.945
Cramer’s V	0.706	0.961	0.961
Pearson correlation coefficient		0.901	

## Results

Our study cohort was composed of *n* = 308 KD cases and *n* = 326 controls (Fig. 1). The median age of KD cases during the acute phase of the disease was 30 months. On the day that the data collection was completed (March 22, 2017), the median age of KD cases was 6.5 years, and 63% were male. Control cases had a median age of 6.3 years on the day of the data collection, and 58% were male (Table 2). KD and control cases were reported from all regions of Germany (Fig. 3). Ethnic background was available from 95.1% of KD cases (*n* = 293) and showed a breakdown of 97.6% Caucasian (*n* = 286), 1.7% Asian (*n* = 5) and 0.7% black African (n = 2).

**Table 2:** Clinical details of KD vs. control cases

	KD cases	Control cases
	(*n* = 308)	(*n* = 326)
Age in years^a^		
Median	6.5	6.3
Sex, n (%)		
Female	114 (37)	137 (42)
Male	194 (63)	189 (58)
KD diagnosis, n (%)		
Complete	269	n.a.
Incomplete with laboratory changes ^b^	15	n.a.
Incomplete with CAA	24	n.a.
Days until start of IVIG therapy		
Average	6.6	n.a.
Min – max	0–32	n.a.
Refractory to IVIG treatment ^c^		
Yes > 1 IVIG cycle	29 (13.4%)	n.a.
Therapy with steroids, n (%)		
Yes, for KD treatment	53 (17.2)	n.a.
Yes, for other reasons	9 (2.9)	n.a.
CAA in acute phase, n (%)		
Yes	36 (11.6)	n.a.
CAA after one year ^c^, n (%)		
Yes	13 (5.9)	n.a.

^a^at the end of the case-control study survey (i.e, March 22, 2017)

^b^ according to the guidelines of the American Heart Association at least three other abnormal supplemental laboratory findings, namely (1) increased alanine transaminase, (2) albumin ≤3.0 g/dL, (3) leukocyturia, (4) anaemia for age, (5) leukocytosis (≥15,000/mm^3^) (6) thrombocytosis (≥450,000/mm^3^) [16]

^c^for cases reported in 2013/2014 *n* = 217; CAA = coronary artery aneurysma; n.a. = not applicable

### Perinatal risk factors compared in KD cases vs. control cases

The dataset matching was prepared for optimal comparison. Thus, only complete case-control pairs (maximum *n* = 227) were used for the conditional logistic regression. When information was missing for one risk factor, the case-control pair was excluded from these analyses. As a sensitivity analysis, we also applied the unconditional logistic regression (maximum *n* = 542). In cases where the results differed from those of the conditional logistic regression, we discussed the discrepancy. For simple description, the entire data set (*n* = 308 KD cases and *n* = 326 control cases) was taken into consideration.

Among the perinatal factors, there were no significant differences between KD and control cases regarding week of gestation, birth weight and parturition (Table 3). However, breastfeeding and vitamin D supplementation did show a protective effect for KD (Table 3). The OR for breastfeeding for longer than two weeks was 0.471 [95% CI 0.260–0.853] as compared to breastfeeding for less than two weeks.

**Table 3:** Demographic characteristics of KD and control cases and potential perinatal factors for KD development (frequencies, means and logistic regression with *p*-value, OR and 95% CI)

	KD cases (*n* = 308)	Control cases (*n* = 326)	Conditional logit – model (*n* = 454) *Unconditional logit - model (n = 542)*	
			OR [95% CI]	*p*-value
Duration of pregnancy in weeks				
Average (±SD)	39 (±2.057)	39 (±1.944)	0.992 [0.891–1.105] *0.965 [0.883–1.050]*	0.891 *0.414*
Parturition^a^, n (%)				
Vaginal	128 (65.6)	194 (70.8)	1 (reference)	0.388 *0.302*
Cesarean section	67 (34.4)	80 (29.2)	1.286 [0.727–2.274] *1.259 [0.813–1.953]*	
Birth weight in gram				
Average (±SD)	3340 (±527.052)	3383 (±537.734)	0.999 [0.999–1.000] *0.999 [0.999–1.000]*	0.715 *0.399*
Range	1560 – 4960	1171 – 4910		
Duration of breastfeeding, n (%)				
< 2 weeks	66 (21.4)	30 (9.2)	1 (reference)	0.013 *0.001*
> 2 weeks^b^	242 (78.6)	296 (90.8)	0.471 [0.260–0.853] *0.415 [0.243–0.687]*	
Vitamin D supplementation in the first year of life, n (%)				
No	64 (20.8)	53 (16.3)	1 (reference)	0.782 *0.051*
Yes	244 (79.2)	273 (83.7)	0.926 [0.537–1.595] *0.623 [0.383–0.995]*	
Duration of vitamin D supplementation in the first year of life in months				
Mean (±SD)	5.16 (±5.749)	6.03 (±5.596)	0.964 [0.931–0.998] *0.965 [0.935–0.995]*	0.039 *0.024*
Regularity of vitamin D supplementation^c^, n (%)				
Irregular	65 (26.6)	55 (20.0)	1 (reference)	0.042 *0.060*
Regular	179 (73.4)	220 (80.0)	0.559 [0.319–0.980] *0.642 [0.402–1.016]*	

^a^ not asked in *n* = 52 control cases vs. *n* = 113 KD cases, ^b^ includes partially breastfed children. ^c^refers only to cases that received vitamin D

The evaluation of vitamin D supplementation also revealed small but significant differences between the KD group and the control group. Our analysis showed that KD cases received Vitamin D for significantly shorter time periods than did the controls (Mean _cases_ = 5 months versus Mean _controls_ = 6 months, OR 0.96, 95% CI 0.931–0.998, *p* = 0.039, see Table 3).

To estimate a possible confounding, the association between breastfeeding and vitamin D supplementation was tested. The measurements of association showed no obvious associations between vitamin D supplementation and breastfeeding: Spearman-Rang-Correlation (r_s_ = − 0.039), Eta-Squared (η^2^ = 0.000) and Cramer’s V (V = 0.141).

### Influence of risk factors on the clinical course

We tested whether the duration of breastfeeding and of vitamin D supplementation influenced the clinical course of KD cases. The clinical variables CAA, being refractory to IVIG treatment, and the level of inflammatory serum markers were analysed to compare KD cases with shorter vs. longer breastfeeding periods and/or longer vs. shorter vitamin D supplementation. Comparing KD cases with CAA (in the acute phase) to KD cases without detection of CAA did not show significant differences with respect to the duration of breastfeeding and vitamin D supplementation. Similarly, KD cases being refractory to IVIG did not differ from IVIG responders (Table 4). Also, the impact on systemic inflammatory serum markers (CRP, leukocytes and platelets) did not demonstrate any significant difference (Table 4).

**Table 4:** Influence of vitamin D supplementation and breastfeeding on the course of disease

	Vitamin D supplementation (*n* = 308)			Breastfeeding (*n* = 308)		
	0–6 months	7–12 months	*p-value* *[95% CI]*	< 2 weeks	> 2 weeks	*p-value* *[95% CI]*
CAA, n (%)						
Yes	13 (11.4)	23 (11.9)	*0.905* *[−0.079–0.070]*	5 (7.6)	31 (12.8)	*0.185* *[− 0.130–0.025]*
No	101 (88.6)	171 (88.1)		61 (92.4)	211 (87.2)	
Refractory to IVIG^a^, n (%)						
Yes	9 (11.5)	20 (14.5)	*0.543* *[−0.125–0.066]*	4 (8.7)	25 (14.7)	*0.233* *[−0.160–0.039]*
No	69 (88.5)	119 (85.5)		42 (91.3)	145 (85.3)	
Laboratory, average (±SD)						
CRP ^b^	93,55 (±86.703)	105,08 (±82.948)	*0,248* *[−31.119–8.057]*	103,04 (±84.499)	100,20 (±84.539)	*0,809* *[−20.255–25.942]*
Thrombocytes ^c^	235.48 (±280.350)	210.95 (±241.805)	*0.421* *[−35.367–84.431]*	570.56 (±277.776)	577.64 (±235.789)	*0.705* *[−54.229–80.070]*
Leukocytes ^d^	17.44 (±7.853)	17.31 (±7.613)	*0.885* *[−1.667–1.931]*	17.23 (±6.582)	17.39 (±7.978)	*0.883* *[−2.279–1.962]*

^a^ only KD patients from 2013/2014 n = 217; ^b^ in mg/dl; ^c^ in T/μl; ^d^ in T/μl

In addition, we investigated whether breastfeeding and vitamin D supplementation might influence disease onset, since not-breastfed/not-supplemented cases potentially might develop KD earlier than breastfed/supplemented cases (Table 5). However, the median age in the two categories (< 2 weeks vs. > 2 weeks of breastfeeding, along with regular vs. irregular supplementation of vitamin D) was not significantly different (*p*-value 0.802 and 0.534, respectively). For the duration of vitamin D supplementation, the correlation coefficient was small and negative. The coefficient of the linear model showed no significant relation to disease onset (Table 5).

**Table 5:** Influence of vitamin D supplementation and breastfeeding on disease onset

	Age in months ^a^ (±SD)	Wilcoxon rank	Spearman correlation coefficient
	(n = 308 ^b^)	p-value	
Regularity of vitamin D supplementation ^c^			
Irregular	34.95 (±24.329)	0.534	
Regular	35.56 (±26.592)		
Duration of vitamin D supplementation in the first year of life in months			
0–6	39.04 (±26.90)		−0.095
7–12	34.95 (±26.07)		
Duration of breastfeeding ^d^			
< 2 weeks	36.14 (±23.691)	0.802	
> 2 weeks ^c^	36.51 (±27.093)		

^a^ at onset of fever, ^b^ only KD cases, ^c^ refers only to cases that got vitamin D (*n* = 244), ^d^ includes partially breastfed cases

## Discussion

Our study is the first of a nationwide, population-based cohort in Germany. The study investigates various perinatal and infancy factors in association with Kawasaki disease and its disease course. Our data reinforce the findings of a large study from Japan which has indicated that breastfeeding may be protective for KD [14]. Vitamin D supplementation appears to have a minor protective effect for KD as well. However, an influence of these factors on the clinical course (development of CAA, raised systemic inflammatory markers, being refractory to IVIG treatment) was not observed. Since the risk factors age, sex and place of residence were used as matching factors, they could not be included in our analysis of potential risk factors.

Our data regarding vitamin D supplementation showed that KD cases in our cohort received significantly less vitamin D than their healthy controls. In a recently published study that measured vitamin D levels during the acute phase of KD, levels proved to be significantly lower compared to controls [18]. Stagi et al. also have described a reduced serum vitamin D concentration in KD cases. These authors suggested that vitamin D may be reduced by the inflammatory process [19]. Our findings indicate that lower vitamin D levels may already play a role before disease onset, since in our cohort, children with KD had a shorter duration of vitamin D supplementation. However, due to our study design, we were not able to determine the actual vitamin D levels prior to the development of KD.

Because the skin of infants is not able to produce sufficient vitamin D, without supplementation, infants will develop vitamin D deficiency [20]. For this reason, in countries such as the USA, infant food is supplemented with vitamin D. In countries such as Germany, a daily vitamin D supplementation is recommended during the first year of life [8]. The Nutrition Commission of the German Society for Pediatric and Adolescent Medicine recommends 400–500 IU / day during the first year of life [21].

The anti-inflammatory and immunomodulatory effects of vitamin D are well-described in several studies [22]. Vitamin D appears to block factors (e.g., tumor necrosis factor α) that are essential for the activation of proinflammatory cytokines [23]. This raises the possibility of vitamin D being used as complementary therapy in immune-mediated diseases such as KD. Some studies already indicate that reduced vitamin D levels may negatively effect the clinical course. First, reduced vitamin D levels seem to be associated with the prevalence of cardiovascular diseases and the development of CAA [19, 24]. Low vitamin D levels inducing endothelial dysfunction may partially explain this association [25]. Second, Zhang et al. have described significantly reduced vitamin D levels in KD patients who were refractory to IVIG treatment [18]. We investigated both of these factors — CAA development and being refractory to IVIG treatment — but were not able to find a significant association with vitamin D supplementation. Furthermore, due to the anti-inflammatory activity of vitamin D, we also investigated other inflammatory serum markers, including CRP, thrombocytes and leukocytes — ones that might indicate higher disease activity in KD cases. However, the markers did not differ between KD cases with longer vs. shorter vitamin D supplementation. Stagi et al. have reported on a negative association regarding low vitamin D serum levels with CRP titers [19]. Additionally, Jun et al. retrospectively reviewed whether low vitamin D levels were associated with resistance to IVIG therapy in KD cases. They found vitamin D deficiency to be associated with IVIG resistance, but not associated with inflammatory markers [26]. In summary, we conclude that vitamin D could potentially play a role in the inflammatory process of KD. However, for a better understanding of its mechanism, additional prospective studies would be necessary.

The perinatal factor of breastfeeding showed a significant protective effect. In general, breastfeeding is recommended for at least six months due to its various health benefits [27]. Breast milk contains lactoferrin and lysozyme, which non-specifically inhibit all types of pathogenic microorganism [28]. In addition, secretory immunoglobulin A confers specific protection against mucosal pathogens [29, 30]. Breastfeeding rarely has been considered in association with KD. Data from a recent study in Japan showed that children who were breastfed were less likely to develop KD [14]. The Japanese authors described a protective effect for both exclusive (OR 0.26; 95% CI 0.12–0.55) and partial breastfeeding (OR 0.27; 95% CI 0.13–0.55) as compared to formula milk [14]. Even partially breastfed children showed a general benefit. Therefore, the protective effect of breast milk probably is not linked to the duration of breastfeeding but rather to substances in either breast milk or colostrum [14], the latter of which contains high levels of secretory antibodies [31]. Additional investigations will be needed to identify these protective substances. In the future, infants who cannot be breastfed might be able to receive these protective substances for protective purposes.

In general, breastfeeding has a positive effect on the outcome of infections in infants. For example, Nishimura et al. have described shorter hospital stays and better outcomes in breastfed children infected with respiratory syncytial virus [32]. However, the effect of breastfeeding on the outcome of KD remains unknown. In our study, we were unable to find an association between the duration of breastfeeding and the appearance of CAA, being refractory to IVIG treatment or the amount of inflammatory serum markers. Therefore, prolonged breastfeeding seems not to be associated with a milder clinical course in KD. Protection against infection by breast milk, the so-called maternal passive immunity, relates to the first six months of feeding [33]. We investigated the potential impact of breastfeeding and vitamin D supplementation on the age of disease onset. However, in shorter-breastfed and/or shorter-supplemented cases, KD did not occur sooner than in other KD cases. It is therefore questionable whether maternal passive immunity has an effect on the age of KD onset.

In conclusion, breastfeeding seems to have a protective effect on the development of KD, supporting the health benefit of general breastfeeding recommendations.

The strength of our case-control study is based upon the large number of KD cases in a Germany-wide cohort, along with the excellent comparability between our KD and control groups. KD cases were reported from all regions of Germany, as were the control cases who were recruited in parallel. A possible bias by the confounder age, sex and place of residence was able to be minimized through a thorough matching process [34]. In addition, all KD cases were evaluated using a standardized questionnaire and they fulfilled internationally-accepted KD criteria [16]. These strict criteria minimized the risk of misdiagnosed KD cases being included in our study. However, the validity of our results should be interpreted in light of our retrospective study design and considered in context of its known limitations. Although KD cases have been reported as part of a prospective surveillance study, data analysis for this study was based upon a retrospective survey conducted over a period of three years. The time period between the acute phase of KD and that of this survey was at least one year. Data quality strongly depends upon the memory ability of the parents, the so-called recall bias. Therefore, we cannot rule out the possibility that families of sick children may have been more likely to remember potential risk factors and/or living conditions than families in the control group did. Another limitation relates to the fact that KD cases were reported from a large number of hospitals, most of whom were without a standardized treatment protocol. KD therapy varies considerably. Some of our KD cases received corticosteroids early in the course of disease that might have impacted the patients’ clinical course, potentially outweighing the influence of vitamin D supplementation and breastfeeding. Finally, a potential confounding between different variables cannot be ruled out. For example, a higher social status often determines a healthier life style. By recruiting control cases via friends and relatives of KD cases, an attempt was made to achieve the best possible comparison regarding socio-economic status. Due to data protection regulations, we were not able to collect additional data regarding the socio-economic status of both KD and control cases.

Another limitation relates to the simultaneous screening for several risk factors. The so-called multiple testing problem (look-elsewhere effect) describes randomly significant findings due to the large number of factors studied [35]. For this reason, our findings should be verified in future prospective studies of larger and more diverse study populations.

## Conclusion

In association with KD in our study population, our results indicate protective effects of vitamin D supplementation and further reinforce breastfeeding to have a protective effect. However, these factors seem not to influence the natural course of the disease. Although the effects were small, they nevertheless underscore the overall benefit of both interventions.

## Additional files

Additional file 1: Questionnaire Kawasaki Disease: ESPED (population-based german pediatric surveillance study). (PDF 146 kb)

Additional file 2: Questionnaire Kawasaki Disease: Perinatal risk factors. (PDF 160 kb)

## Abbreviations

95% CI: 95% Confidence interval
CAA: Coronary Artery Aneurysm
e.g.: for example
ESPED: Nation-wide population-based German Pediatric Surveillance Study
ESR: Erythrocyte sedimentation rate
IVIG: Intravenous immunoglobulin
KD: Kawasaki Disease
OR: Odds Ratio
