# Supplementary material for: Breastfeeding and vitamin D supplementation reduce the risk of Kawasaki disease in a German population-based case-control study
Source: BMC Pediatr. 2019 Feb 26;19:66. doi: 10.1186/s12887-019-1438-2 (PMC6390341; doi:10.1186/s12887-019-1438-2)
Supplement: Supplementary file 1 — Questionnaire Kawasaki Disease: ESPED (population-based german pediatric surveillance study). (PDF 146 kb) [file 12887_2019_1438_MOESM1_ESM.pdf]

Erhebungseinheit für seltene pädiatrische Erkrankungen in Deutschland

POPULATION-BASED GERMAN PEDIATRIC SURVEILLANCE STUDY

Kawasaki Disease (KD) in Germany

ESPED
Arbeitsgruppe am KKS
Heinrich-Heine-Universität Düsseldorf
z.Hd. Frau Heinrich
Postfach 10 22 44
40013 Düsseldorf

Clinic: *(Hospital's stamp if available)*

ESPED-NR:
Reporting month:
Date:

Reporting Pediatricians:
Hospital:...
Name:...
Phone.:...
E-Mail:...

Case:

Initials of the case: I_X_I___I I_X_I___I (*Surname* / *First Name*)

Sex: O Male O Female

Date of birth: I_X_ I____I______I (*Month* / *Year*)

Postal code I___I___I___I_X_I_X_I (*First three numbers*)

Nationality: O German O Others: ________________________

Ethnic background: ____

Weight: ______ kg Height: ________ cm

Clinical Course

Diagnosis: O Complete Kawasaki Disease O Incomplete Kawasaki Disease

O Fever >38,5°C:	1st occurence I____I____I______I *(dd I mm I jjjj)*	Duration: ______ Days	O No fever
O Conjunctival injection:	1st occurence I____I____I______I *(dd I mm I jjjj)*	O Unilateral O Bilateral	Exudative: Yes / No
O Lymphadenopathy:	1st occurence I____I____I______I *(dd I mm I jjjj)*	O Unilateral O Bilateral	
O Exanthema:	1st occurence I____I____I______I *(dd I mm I jjjj)*	O Monomorphous O Polymorphous	
O Desquamation:	1st occurence I____I____I______I *(dd I mm I jjjj)*		
O Enanthema of lips and oral cavity:	1st occurence I____I____I______I *(dd I mm I jjjj)*		
O Changes in extremities:	1st occurence I____I____I______I *(dd I mm I jjjj)*		

Diagnostic Criteria

**Laboratory values :**	Not determined	Value	Date (dd.mm.jjjj)
Albumin	O	g/dl	
Alanine Aminotransaminase	O	U/ll	
Aspartate Aminotransferase	O	U/l	
CRP	O	mg/l	
Thrombocytes	O	T/µl	
Haemoglobin	O	g/dl	
Leukocytes	O	T/µl	

Leukocyturia O Not determined O No O Yes Date: I____I____I______I *(dd I mm I jjjj)*
ESR: ____/_____ (mm/h), Date: I____I____I______I *(dd I mm I jjjj)*

Echocardiography: O No O Yes, Date: I____I____I______I *(dd I mm I jjjj)*
Coronary aneurysm: Yes/ No Diameter: ________ mm
LCA: ___________ LAD: ___________ LCX: ___________ RCA: ____________

Therapy

ASS (Initial: high-dose): Dose: __________ mg/kg body weight/d Start: I____I____I_______I *(dd I mm I jjjj)*
(Duration: low-dose): Dose: __________ mg/kg body weight/d Start: I____I____I_______I Duration _____ weeks

Intravenous Immunoglobulin (IVIG): Total amount. ____ O No IVIG, because:_______________________

1st Dose: Dose: ______ g/kg body weight/d, Date I___I___I_____I *(dd I mm I jjjj)*

2nd Dose: Dose: ______ g/kg body weight/d, Date I___I___I_____I *(dd I mm I jjjj)*

Following: Dose: ______ g/kg body weight/d, Date I___I___I_____I Dose: ______ g/kg body weight/d, Date I___I___I_____I

Steroids: for KD treatment O Yes O No, for other reasons:_______________________________

Name: _____________________ Dose: ______________ Start: I___I___I_____I Duration_________Days

OUTCOME

O Cure O Recurrence: _____(Number) O Deceased: ____________ (Date)

THANK YOU FOR YOUR SUPPORT
