# Supplementary material for: Breastfeeding and vitamin D supplementation reduce the risk of Kawasaki disease in a German population-based case-control study
Source: BMC Pediatr. 2019 Feb 26;19:66. doi: 10.1186/s12887-019-1438-2 (PMC6390341; doi:10.1186/s12887-019-1438-2)
Supplement: Supplementary file 2 — Questionnaire Kawasaki Disease: Perinatal risk factors. (PDF 160 kb) [file 12887_2019_1438_MOESM2_ESM.pdf]

Questionnaire Kawasaki Disease

Perinatal risk factors

Attention: The questionnaire cannot be cached.
It will take approximately 10 minutes:
ESPED Nr.: ____________

Characteristics of perinatal period

- Duration of pregnancy (in weeks): ______________
  e.g.: 38+2 = 38 weeks plus 2 days (please document 39)
  e.g.: 1 week longer = 41 weeks (please document 41)

- Birth weight (in gramm)

- Parturition?
  - Cesarean Section
  - Vaginal

- How long did you breastfeed your child?
  - < 2 weeks
  - > 2 weeks

- Vitamin D supplementation in the 1st year of life?
  - Yes
  - No

- How long did you supplement Vitamin D (in months): ___________

- How often did you supplement vitamin D?
  - Daily
  - Every second day
  - Not on a daily basis

Family and Environment

- Who is doing a vegetarian diet?
  - None
  - Mother
  - Father
  - Child

- Did you have pets (during the acute phase of the child suffering from Kawasaki Disease)
  - Yes
  - No

  If yes, which kind of:___________________

- Number of persons in household (during the acute phase of the child suffering from Kawasaki Disease): ____________

- Number of children (at the time of suffering from Kawasaki Disease): ___________

- Living space in m² (at the time of suffering from Kawasaki Disease): _____________

- Do you have any additional remarks: ____

End

Thank you for your time and your support!
